# Sirt7/HIC1 complex participates in hyperglycaemia‐mediated EndMT via modulation of SDC1 expression in diabetic kidney disease and metabolic memory

**DOI:** 10.1111/jcmm.18336

**Published:** 2024-04-30

**Authors:** Lihong Lu, Minmin Zhu, Qichao Wu, Zhirong Sun, Xiangyuan Chen, Changhong Miao

**Affiliations:** ^1^ Department of Anesthesiology, Zhongshan Hospital Fudan University Shanghai China; ^2^ Department of Anesthesiology Fudan University Shanghai Cancer Center Shanghai China; ^3^ Department of Oncology, Shanghai Medical College Fudan University Shanghai China; ^4^ Department of Anesthesiology, Shanghai General Hospital Shanghai Jiao Tong University School of Medicine Shanghai China

**Keywords:** diabetic kidney disease, HIC1, Sirt7

## Abstract

Diabetic kidney disease (DKD), a primary microvascular complication arising from diabetes, may result in end‐stage renal disease. Epigenetic regulation of endothelial mesenchymal transition (EndMT) has been recently reported to exert function in metabolic memory and DKD. Here, we investigated the mechanism which Sirt7 modulated EndMT in human glomerular endothelial cells (HGECs) in the occurrence of metabolic memory in DKD. Lower levels of SDC1 and Sirt7 were noted in the glomeruli of both DKD patients and diabetes‐induced renal injury rats, as well as in human glomerular endothelial cells (HGECs) with high blood sugar. Endothelial‐to‐mesenchymal transition (EndMT) was sustained despite the normalization of glycaemic control. We also found that Sirt7 overexpression associated with glucose normalization promoted the SDC1 expression and reversed EndMT in HGECs. Furthermore, the sh‐Sirt7‐mediated EndMT could be reversed by SDC1 overexpression. The ChIP assay revealed enrichment of Sirt7 and H3K18ac in the SDC1 promoter region. Furthermore, hypermethylated in cancer 1 (HIC1) was found to be associated with Sirt7. Overexpression of HIC1 with normoglycaemia reversed high glucose‐mediated EndMT in HGECs. The knockdown of HIC1‐mediated EndMT was reversed by SDC1 upregulation. In addition, the enrichment of HIC1 and Sirt7 was observed in the same promoter region of SDC1. The overexpressed Sirt7 reversed EndMT and improved renal function in insulin‐treated diabetic models. This study demonstrated that the hyperglycaemia‐mediated interaction between Sirt7 and HIC1 exerts a role in the metabolic memory in DKD by inactivating SDC1 transcription and mediating EndMT despite glucose normalization in HGECs.

## 1 INTRODUCTION

Diabetic kidney disease (DKD), as the chief microvascular complication of diabetes, is generally followed by end‐stage renal disease.^1^, ^2^ Despite the efficient control of glycaemia, the diabetes‐related microvascular complications were continued or aggravated as reported recently,^3^, ^4^ defined as ‘metabolic memory’, which has been identified to exert a vital function in the progression of DKD.^5^ However, the mechanism by which hyperglycaemia induces metabolic memory in DKD is seldomly researched.

The process of endothelial‐to‐mesenchymal transition (EndMT) in endothelial cells is acknowledged as a critical factor in metabolic memory development,^6^ as well as DKD.^7^, ^8^ EndMT involves a complex process of cell differentiation, the endothelial cells are separated and removed from endothelium, losing endothelial characteristics, and then obtain mesenchymal properties. This process is characterized by the deficiency of endothelial markers such as CD31 and the acquisition of mesenchymal traits, involving αSMA,^9^ which is considered as a form of epithelial‐to‐mesenchymal transition (EMT), both may possess uniform modulators.^10^ Syndecan‐1 (SDC1) is the major heparan sulfate proteoglycan on cellular surface.^11^ Studies have indicated that the depletion of SDC1 is implicated in the process of EMT,^12^ while overexpression inhibits it in some situations conversely.^13^ The involvement of SDC1 in hyperglycaemia‐induced EndMT within glomerular endothelial cells remains unexplored.

Recently, a growing body of studies has paid attention to the effect of epigenetics on the pathogenesis of diabetic kidney disease and metabolic memory.^14^, ^15^ Among them, histone modification has been reported to serve as a crucial process of the modulation of gene transcription and participates in the occurrence of metabolic memory and DKD,^15^ which has also been demonstrated in our previous work.^16^, ^17^, ^18^, ^19^ Apart from this, histone acetylation also plays a crucial role in metabolic memory occurrence^20^ and DKD.^21^ Sirtuin 7 (Sirt7) stands out as the sole member of the sirtuin family predominantly situated in the nucleus, where it regulates acetylation of H3K18 (H3K18ac) through its deacetylase activity, thereby playing a crucial role in preserving the integrity of the genome.^22^, ^23^ Additionally, Sirt7 has been demonstrated to exert a regulatory function in EMT and the development of DKD.^24^, ^25^ Herein, we hypothesize that Sirt7 is pivotal in EndMT by influencing SDC1 transcription, contributing to metabolic memory and the progression of DKD. Crucially, we delved into the underlying mechanism through which Sirt7 governs the expression of SDC1.

## 2 MATERIALS AND METHODS

### 2.1 Subjects

This research enlisted 30 individuals diagnosed with diabetic kidney disease (DKD), all of whom were selected in succession. Within this cohort, 15 patients exhibiting suboptimal blood sugar management and elevated levels of glycated haemoglobin were categorized into the DKD cohort. Conversely, the remaining 15 patients, who demonstrated effective blood sugar regulation and standard glycated haemoglobin levels, were grouped into the metabolic memory/diabetic kidney disease category (MD). Additionally, the study included 15 patients with renal tumours but without diabetes and with preserved kidney function as the control group (Con). Prior to initiating the study, all participants were informed of consent. The research complied with the ethical guidelines of the Declaration of Helsinki and obtained the endorsement of the Ethics Committee at Huzhou Central Hospital, under the licence number 20191209‐01. Participant details are summarized in Table 1.

**TABLE 1:** Characteristics of participants (A) and rats (B) in different groups.

(A)			
Human variables	Con	DKD	MD
Age (years)	56 (54, 58)	57 (51, 62)	54 (48, 58)
BMI (kg/m^2^)	21.84 ± 1.39	22.11 ± 1.53	21.64 ± 1.41
SBP (mmHg)	11.80 ± 4.60	134.42 ± 9.29	133.13 ± 6.09
DBP (mmHg)	70.87 ± 3.18	72.33 ± 6.17	67.53 ± 7.03
HbA1C (%)	5.03 ± 0.46	9.19 ± 1.61****	5.13 ± 0.59*
FBS (mmol/L)	6.1 (4.8, 6.2)	8.2 (7.4, 11.3)	6.0 (5.5, 6.9)*
CREA (μmol/L)	60.3 ± 6.54	269.7 ± 81.57****	208.67 ± 32.83****
CCr (mL/min)	109.4 ± 6.81	55.07 ± 14.00****	61.47 ± 9.43****
24hUTP (mg)	117.8 ± 9.09	3336.07 ± 622.84****	3341.8 ± 649.2****
TP (g/L)	67.7 ± 4.59	45.07 ± 11.49****	48.87 ± 5.42****

(B)			
Rat variables	Con	DRI	MD
Weight (g)	377 ± 14.53	530.6 ± 11.14****	522.9 ± 12.43****
Weight of Kidney (g)	1.72 ± 0.27	2.8 ± 0.32**	2.6 ± 0.27**
FBS (mmol/L)	3.9 (3.6, 4.1)	23.3 (21.2, 24.3)	3.9 (3.7, 4.1)
UMP (mg/L)	119 (115, 123)	149 (143, 167)**	148 (145, 151)**
TG (mmol/L)	0.85 ± 0.206	4.1 ± 0.37****	4.0 ± 0.25****
UA (mmol/L)	224 (220, 234)	343 (324, 354)****	333 (289, 345)***
UREA (μmol/L)	4.08 ± 0.25	11.4 ± 0.44****	10.1 ± 0.81****
BUN (umol/L)	6.1 ± 0.62	9.7 ± 1.57***	9.0 ± 1.67***
TC (mmol/L)	1.64 ± 0.41	3.7 ± 0.56***	3.6 ± 0.30***
LDL (mmol/L)	0.47 ± 0.13	2.0 ± 0.21**	1.9 ± 0.15**
HDL (mmol/L)	1.25 ± 0.25	0.91 ± 0.40*	1.0 ± 0.17*

*Note*: Data are presented as the means ± SD or median (lower quartile, upper quartile), *p < 0.05, **p < 0.01, ***p < 0.001, ****p < 0.0001, *n* = 15 per human group, *n* = 10 per rat group.

Abbreviations: 24hU‐TP, 24 h urinary total protein; BMI, body mass index; BUN, blood urea nitrogen; CCr, creatnine clearance; CREA, creatinine; DBP, diastolic blood pressure; FBS, fasting blood sugar; HbA1c, glycated haemoglobin; HDL, high density lipoprotein; LDL, low density lipoprotein; SBP, systolic blood pressure; TC, total cholesterol; TG, triglyceride; TP, total protein; UA, uric acid; UMP, urine microprotein; UREA, urine creatinine.

### 2.2 Rat model

Animal experiments have acquired ethical approval from the animal ethical clearance committee of our institute (Fudan University Shanghai Cancer Center, 2020, JS‐391). In this study, Sprague Dawley rats within 300–400 g were involved in experiments. All operations adhered to the guidelines provided by Fudan University Shanghai Cancer Center's Guide for the Care and Use of Laboratory Animals, as well as the US NIH's Guide for the Care and Use of Laboratory Animals (2011 edition). Following unilateral nephrectomy performed under isoflurane anaesthesia (3%–4% for induction, 1.5%–2.5% for maintenance, with 100% oxygen), the rats were returned to their housing for a recovery period of 9 weeks. At 3 weeks post‐nephrectomy, the control group (*n* = 10) were administered a one‐time intraperitoneal dose of citrate buffer (0.1 M, pH 4.5). Another set of rats was subjected to a high‐glucose and high‐fat diet for 9 weeks post‐nephrectomy, along with a single streptozotocin (50 mg/kg, i.p.) 3 weeks after the nephrectomy, and this group was labelled as the diabetes‐induced renal injury group (DRI, *n* = 10). The DRI group, intended to examine the impact of glycaemic normalization, was administered subcutaneous insulin (1 IU/100 g body weight) during the final 3 weeks, thus being categorized as the metabolic memory/diabetes‐induced renal injury group (MD, *n* = 10). To investigate the potential protective role of Sirt7 against metabolic memory in diabetic kidney disease, MD rats were injected with an adeno‐associated virus containing the full‐length rat Sirt7 gene (AAV‐Sirt7, GeneChem Co. Ltd., Shanghai, China) or a control vector (AAV‐GFP, GeneChem Co. Ltd., Shanghai, China) into the remaining kidney at the time of nephrectomy. These rats were subsequently identified as either the MD + AAV group or the MD + AAV‐Sirt7 group.

### 2.3 Collection of rat blood samples and urine specimen

Prior to euthanasia, the rats' 24‐h urine collections were finished in metabolic cages. Additionally, bloods were sampled from the rats. These plasma and urine specimens were then stored at a temperature of −80°C pending for further analysis. The measurement of serum creatinine concentrations was performed utilizing a creatinine assay kit (Jiancheng Biology, Nanjing, China). To determine serum urea nitrogen (BUN) levels, a BUN test kit (Jiancheng Biology, Nanjing, China) was employed. The quantification of albumin in 24‐h urine samples was carried out using an advanced BCA protein assay kit sourced (Beyotime, Shanghai, China).

### 2.4 Immunohistochemistry (IHC)

Participant samples and rat kidney tissues were embedded in paraffin before undergoing immunohistochemistry (IHC) referring to the provided instructions. The paraffin‐embedded sections received the primary antibodies against hypermethylated in cancer 1 (HIC1) (Abclonal, Wuhan, China), Sirt7 (ProteinTech, Wuhan, China), SDC1 (Abclonal, Wuhan, China), CD31 (ProteinTech, Wuhan, China), and αSMA (ProteinTech, Wuhan, China). This incubation took place at 4°C overnight within a moisture‐controlled chamber. Following this, the sections were exposed to a secondary antibody at ambient temperature for 90 min and then developed using a DAB Detection Kit (GeneTech, Shanghai, China).

### 2.5 Cell culture and reagents

Dulbecco's Modified Eagle Medium (DMEM) supplemented with 5 mM glucose was utilized for HGECs (Procell, Wuhan, China) cultured, with 10% fetal bovine serum, and penicillin/streptomycin (100 μg/mL) at 37°C in a humid environment enriched with 5% CO_2_. For the high glucose (HG) treatment, the cells underwent three phosphate‐buffered saline washes to eliminate any residual complete medium and were then incubated for an additional 6 days in DMEM enriched with 25 mM glucose, 10% fetal bovine serum, and penicillin/streptomycin (100 μg/mL). The metabolic memory (MM) group cells were initially cultured in DMEM containing 25 mM glucose, 10% fetal bovine serum, and penicillin/streptomycin (100 μg/mL) for 3 days, followed by a 3‐day culture period in DMEM with 5 mM glucose. An osmotic control was established using 5 mM glucose combined with 20 mM mannitol.

### 2.6 Western blot

Whole‐cell lysates were produced using a cell lysis solution from Cell Signalling Technology (Danvers, MA). The proteins were denatured by heating in loading buffer at 100°C for 10 min. Equal amounts of protein (50 μg) from all HGEC groups were separated using 8%–10% SDS‐PAGE and subsequently transferred onto PVDF membranes. These membranes were then blocked using a protein‐free rapid‐blocking solution (Beyotime Biotechnology, China) for 1 h. After the blocking step, the membranes underwent overnight incubation with designated primary antibodies at 4°C. The antibodies utilized in this investigation included anti‐β‐actin, anti‐HIC1, and anti‐Sirt7; anti‐SDC1, anti‐H3K18ac, anti‐CD31, anti‐αSMA, anti‐Snail, anti‐ERK1/2, and anti‐phospho‐ERK1/2 (ProteinTech, Wuhan, China). After undergoing five washes, the membranes were treated by secondary antibodies at ambient temperature for an hour, followed by another five washes with PBST. The protein signals were detected using an ECL system (Beyotime Institute of Biotechnology, Shanghai, China).

### 2.7 RNA extraction and quantitative real‐time PCR (qPCR)

RNA was extracted using the EZ‐press RNA Purification kit (EZBioscience). For the synthesis of cDNA necessary for quantitative PCR (qPCR), the Hifair® II 1st Strand cDNA Synthesis (SuperMix by Yeasen, Shanghai, China) was utilized. Following the initial steps, qPCR was performed with Hieff UNICON® qPCR TaqMan Probe Master Mix (Yeasen, Shanghai, China) using an ABI7500 Real‐Time PCR system (Applied Biosystems). The primers used in this research are listed in Table S1.

### 2.8 Co‐immunoprecipitation (CoIP)

Cell lysis buffer containing PMSF and protease inhibitor was utilized to extract 30 μL cell protein from HGECs, which was then boiled in the loading buffer at 100°C for 10 as the input. For the internal immunoprecipitation process, cellular extracts were combined with appropriate primary antibodies and 50 μL of protein A/G Dynabeads, followed by a 12‐h incubation at 4°C. The mixture was centrifugated at 14,000 rpm for 5 s for precipitate and washed three times with a pre‐cooled Washing Buffer. Subsequently, 10 μL sample from the input, IgG, and IP fractions were utilized for conducting Western blot analysis.

### 2.9 GST pull‐down assay

His‐Sirt7 (ProteinTech, Wuhan, China) and GST‐HIC1 (Novus Biologicals) fusion protein were used to perform GST pulldown assay. In brief, the fusion protein was mixed in a GST or His buffer at 4°C for 12 h. Then, anti‐His or anti‐GST beads were added for incubation with fusion proteins for more than 4 h. The magnetic beads were washed three times and then utilized for Western blot analysis.

### 2.10 Immunofluorescence (IF)

HGECs were inoculated in confocal dishes. After reaching suitable density, the cells underwent fixation with 4% paraformaldehyde for 15 min, followed by permeabilization with 0.3% Triton X‐100 for 5 min, and were then blocked at room temperature for 1 h. Subsequently, they were mixed with an anti‐Sirt7 antibody (Abcam, Cambridge, UK), and an anti‐HIC1 antibody (Abclonal, Wuhan, China) for incubation at 4°C overnight. Then the cells underwent three PBS washes before being exposed to a fluorescent secondary antibody at ambient temperature for 1 h. Nuclei staining was performed using 4,6‐diaminophenyl indole (DAPI). A confocal fluorescence microscope (Leica, Solms, Germany) was employed to capture the images.

### 2.11 siRNA, shRNA and Sirt7 mutant plasmid

HGECs were transfected with sh‐Sirt7, si‐HIC1, si‐SDC1, an SDC1 overexpression plasmid, a HIC1 overexpression plasmid, a Sirt7 overexpression plasmid and a Sirt7 mutant plasmid using Lipofectamine 3000 (Invitrogen, USA). The sequences of sh‐Sirt7 (Biotend, Shanghai, China) were shRNA‐a, 5′‐CCAAATACTTGGTCGTCTA‐3′, and shRNA‐b, 5′‐GAAAGGGAGAAGCGTTAGT‐3′. The sequences of si‐HIC1 (Biotend, Shanghai, China) were siRNA‐a, 5′‐CCCUACCCAUGCACCAUCUdTdT‐3′, and siRNA‐b, and 5′‐AGAUGGUGCAUGGGUAGGGdTdT‐3′. The sequences of si‐SDC1 (Biotend, Shanghai, China) were siRNA‐a, 5′‐GCAGGACUUCACCUUUGAAdTdT‐3′ and siRNA‐b, 5′‐UUCAAAGGUGAAGUCCUGCdTdT‐3′.

### 2.12 Chromatin immunoprecipitation (ChIP) assay

Kit (CHIP Kit ab500, Abcam, England) following the provided instructions. Initially, cells (1 × 10^7) were exposed to 1% formaldehyde for 10 min at ambient temperature to enable cross‐linking between DNA and proteins. The cross‐linking was halted by adding 2.5 mmol/L glycine. Chromatin shearing was achieved through ultrasonic waves. The resulting supernatant, after centrifugation, was incubated overnight with antibodies targeting HIC1, Sirt7, H3K18ac or IgG as a control at 4°C. Agarose beads were then used to capture the antibody‐bound complexes. The cross‐links between DNA and proteins were reversed by incubating in a water bath at 65°C for 6 h. PCR was employed to analyse the sequences enriched from the purified DNA. Additionally, to confirm the simultaneous enrichment of Sirt7, H3K18ac, and HIC1 on the same SDC1 promoter region, a re‐ChIP assay was conducted. The oligonucleotide primer sequences for SDC1 used in the study were as follows: p1 with forward primer 5′‐CCACAGAAAAACGCTGCGAA‐3′ and reverse primer 5′‐CCAGATTCTCCCGTACGCTC‐3′; p2 with forward primer 5′‐GGAACACTGGACACTTCCCG‐3′ and reverse primer 5′‐GAGATGGTGCCAAACGACCT‐3′; p3 with forward primer 5′‐GATTATAGGCACCTGCCACC‐3′ and reverse primer 5′‐CGCGATGGCTCACGCCTGTA‐3′; p4 with forward primer 5′‐CTCACACATGCACTCAAGAA‐3′ and reverse primer 5′‐TAGAGACAAGGTTTCACCAT‐3′.

### 2.13 Dual‐luciferase assay

A Promega Dual‐luciferase Assay Kit (Promega Biotech, USA) was utilized to evaluate the effect of HIC1 on the activity of SDC1 promoter. The promoter region of SDC1 was amplified and then inserted into the pGL3‐basic vector, resulting in the creation of the pGL3‐SDC1 construction. The promoter site was mutated. The pGL3‐SDC1 plasmid and pGL3‐SDC1MUT were transfected along with a Renilla luciferase vector into HGECs. The effect of HIC1 on SDC1 promoter activity was assessed. Cells underwent lysis using 1x passive lysis buffer provided by Promega Biotech, based in the USA. To normalize the activity of firefly luciferase, the corresponding Renilla luciferase activity was used as a reference, utilizing the Dual‐Luciferase Reporter Assay System also from Promega Biotech, USA.

### 2.14 Statistical analysis

Statistical evaluation in this research was conducted with the SPSS 22.0 software package (IBM Corp, Armonk, NY, USA). The Student's *t*‐test was utilized for the comparison of two distinct groups. For analyses involving more than two groups, one‐way analysis of variance (ANOVA) and the Chi‐squared test were employed. The results were represented as either mean ± standard deviation (SD) or median (interquartile range), and a *p* < 0.05 indicates statistical significance.

## 3 RESULTS

### 3.1 Occurrence of EndMT and metabolic memory, and decrease of SDC1 expression in DKD patients and diabetes‐induced renal injury rats

**FIGURE 1:**
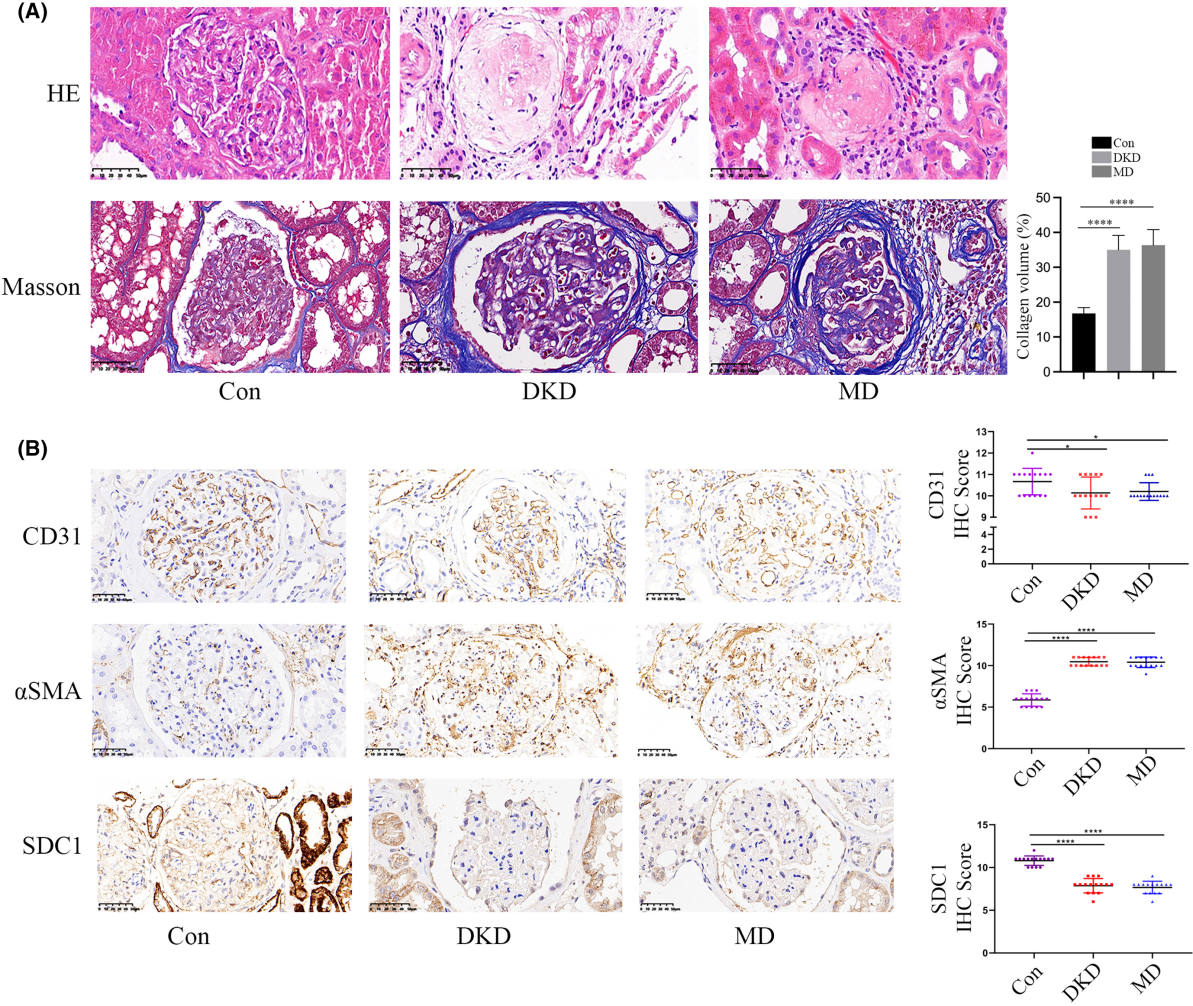
Occurrence of EndMT and inhibition of SDC1 levels persisted even after glycaemia normalization in participants. (A) Representative images of HE staining and Masson's staining of renal biopsy specimens from the participants. (B) The IHC results of CD31, αSMA and SDC1 in renal biopsy specimens of participants. The reduction of CD31 and SDC1 expression, and the increase of αSMA persisted after glycaemia normalization in participants. (*p < 0.05, **p < 0.01, ***p < 0.001, ****p < 0.0001, statistical analysis was carried out by a one‐way ANOVA followed by Bonferroni‐corrected pairwise comparisons for over two groups.)

The details of the DKD and MD patients, along with the control group participants, are presented in Table 1. Figure 1 displays the outcomes of HE and Masson staining of renal biopsy samples from the participants. Masson staining revealed increased blue staining in the renal biopsies of DKD patients, even after glucose levels were normalized, signifying persistent collagen deposition and extensive interstitial fibrosis despite achieving glucose control (Figure 1A). EndMT is suspected to exert a role in the occurrence of metabolic memory^6^ and interstitial fibrosis in DKD.^7^, ^8^ We detected CD31 and αSMA expression in the glomerulus of participants to validate the role of endothelium and found that CD31 expression was decreased, and αSMA expression was increased in glomerulus of DKD and MD patients (Figure 1B) compared to the control. To explore the involvement of SDC1 in modulation of EndMT in DKD we detected SDC1 expression in glomerulus of DKD and MD patients and found the decreased expression of SDC1 in glomerulus of DKD and MD participants (Figure 1B), which is consistent with previous work.^12^, ^13^

The information on the rats employed in this study is listed in Table 1. The results of HE and Masson staining in renal biopsy specimens are shown in Figure S1A. IHC analysis showed that the expression of αSMA was increased, and the expression of CD31 and SDC1 were decreased in kidney of DRI and MD rats (Figure S1B). These data suggested that EndMT and SDC1 participated in diabetes‐induced renal injury and metabolic memory.

### 3.2 High glucose‐mediated EndMT participated in metabolic memory in HGECs via inhibition of SDC1 expression

**FIGURE 2:**
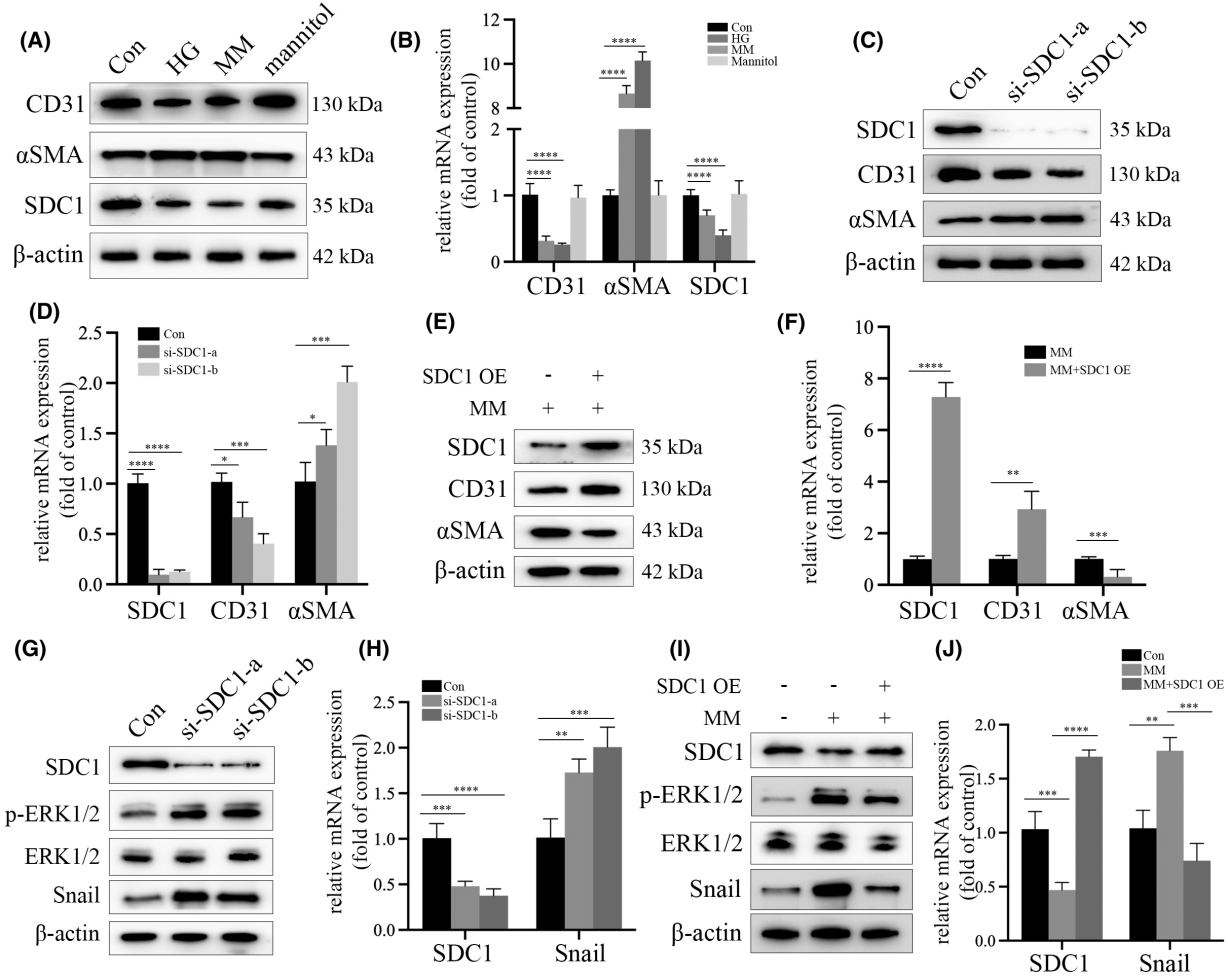
EndMT continued in hyperglycaemic HGECs after glucose normalization, which was inhibited by upregulation of SDC1 expression. (A) Results from the Western Blot analysis indicated that the reduction of CD31 and SDC1 expression persisted, and the increasing trend of αSMA levels continued even after glucose normalization in HGECs. (B) mRNA expression of CD31, αSMA and SDC1 detected by qPCR with corresponding treatment (*n* = 5 times/group). The results indicated the preserved EndMT in hyperglycaemic HGECs even after glucose normalization. (C) Results from the Western Blot analysis indicated that si‐SDC1 inhibited CD31 expression, while increased αSMA levels in HGCEs. (D) Results from the qPCR analysis indicated that CD31 expression was decreased, and αSMA levels were increased in si‐SDC1‐treated HGECs. (E) Results from the Western Blot analysis indicated that upregulation of SDC1 expression increased CD31 expression and decreased αSMA levels in MM‐treated HGECs. (F) Results from the qPCR analysis indicated that SDC1 overexpression increased CD31 expression and decreased αSMA levels in MM‐treated HGECs. (G) Results from the Western Blot analysis indicated that si‐SDC1 increased ERK phosphorylation and Snail expression in HGECs. (H) Results from the qPCR analysis indicated that si‐SDC1 increased Snail expression in HGECs. (I) Results from the Western Blot analysis indicated that upregulation of SDC1 expression decreased ERK1/2 phosphorylation and Snail levels in MM‐treated HGECs. (J) Results from the qPCR analysis indicated that SDC1 upregulation decreased Snail levels in MM‐treated HGECs. (*p < 0.05, **p < 0.01, ***p < 0.001, ****p < 0.0001, statistical analysis was carried out by a one‐way ANOVA followed by Bonferroni‐corrected pairwise comparisons for over two groups.)

The decline in SDC1 expression prompted us to investigate its underlying mechanism in high glucose‐induced EndMT. We therefore constructed the in vitro hyperglycaemia cell model using human glomerular endothelial cells (HGECs) as presented above. The result demonstrated HG inhibited CD31 and SDC1 expression, while increased αSMA expression, even after glucose normalization (Figure 2A,B). Mannitol treatment made no efficiency on CD31, αSMA and SDC1 expression (Figure 2A,B). Moreover, the inhibition of SDC1 expression could induce EndMT in HGECs (Figure 2C,D). Furthermore, overexpression of SDC1 reversed EndMT in HGECs in MM group (Figure 2E,F). These results illustrated that high glucose participated in metabolic memory in HGECs by mediating EndMT via inhibition of SDC1 expression.

To further explore how SDC1 mediated EndMT in HGECs, we detected extracellular signal‐regulated kinase (ERK)/Snail pathway. The results indicated that si‐SDC1 upregulated ERK phosphorylation and Snail expression in HGECs (Figure 2G,H), while SDC1 overexpression inhibited MM‐induced ERK phosphorylation and Snail expression (Figure 2I,J), illustrating that SDC1 mediated EndMT via modulate ERK/Snail pathway.

### 3.3 Sirt7 functions in hyperglycaemia‐induced EndMT via modulation of SDC1 expression in HGECs

**FIGURE 3:**
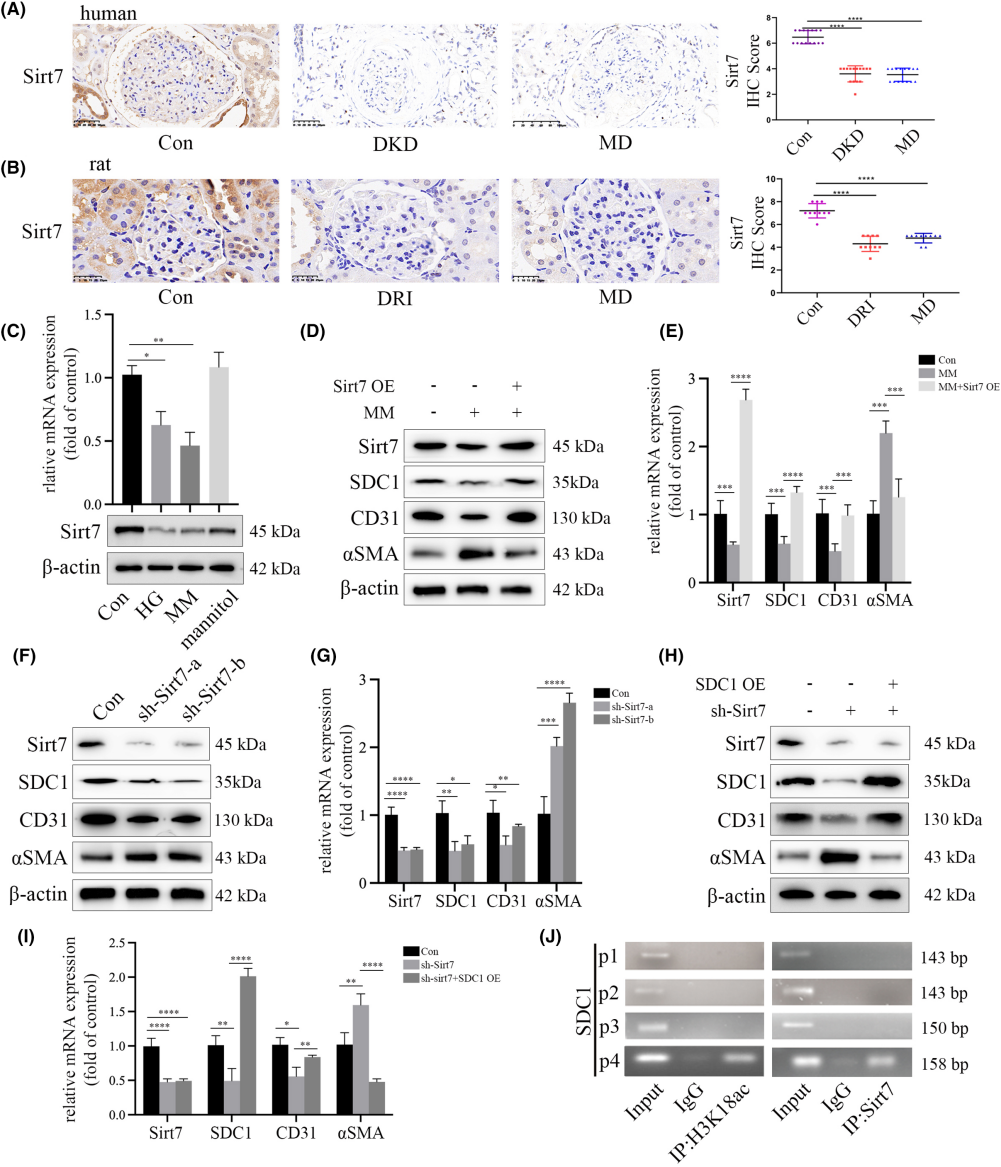
Sirt7 participated in hyperglycaemia‐induced EndMT via modulation of SDC1 expression in HGECs. (A) The IHC results of Sirt7 in renal biopsy specimens of participants. Our data indicated that the reduction of Sirt7 expression persisted even after glycaemia normalization in participants. (B) The IHC results of Sirt7 in renal biopsy specimens of rats. Our data indicated that the reduction of Sirt7 expression persisted even after glycaemia normalization in glomeruli of the rats. (C) The Western Blot analysis and qPCR analysis indicated that the reduction of Sirt7 expression persisted continued even after glucose normalization in HGECs. Results from the Western Blot analysis (D) and qPCR analysis (E) indicated that upregulation of Sirt7 expression increased CD31 and SDC1 expression, and decreased αSMA levels in MM‐treated HGECs. Results from the Western Blot analysis (F) and qPCR analysis (G) indicated that sh‐Sirt7 decreased the expression of SDC1 and CD31, and increased αSMA levels in HGECs. Results from the Western Blot analysis (H) and qPCR analysis (I) indicated that the effect of sh‐Sirt7 on SDC1, CD31 and αSMA expression was reversed by upregulating SDC1 expression in HGECs. (J) ChIP assay indicated that Sirt7 and H3K18ac were enriched on the promotor region of SDC1. (*p < 0.05, **p < 0.01, ***p < 0.001, ****p < 0.0001, statistical analysis was carried out by a one‐way ANOVA followed by Bonferroni‐corrected pairwise comparisons for over two groups.)

It has been indicated that Sirt7 exerts a role in mediating EMT^24^ and the occurrence of DKD.^25^ The decreased expression of Sirt7 in glomerulus of DKD participants and diabetes‐induced renal injury rats in our study confirmed it, which was preserved even after glycaemia turned to normal (Figure 3A,B). The in vitro experiments came to the same conclusion (Figure 3C). The overexpression of Sirt7 could reverse the MM‐mediated SDC1 reduction and EndMT in HGECs (Figure 3D,E). Furthermore, sh‐Sirt7 inhibited the SDC1 expression and mediated EndMT in HGECs, which was similar to that of HG and MM group (Figure 3F,G), and this tendency was reversed by SDC1 overexpression in HGECs (Figure 3H,I). In addition, ChIP assay demonstrated the enrichment of Sirt7 and H3K18ac on the promotor of SDC1 (Figure 3J). These results illustrated the involvement of Sirt7 in hyperglycaemia‐mediated EndMT via modulation of SDC1 expression, thus inducing metabolic memory in HGECs.

### 3.4 Sirt7 interacted directly with HIC1

**FIGURE 4:**
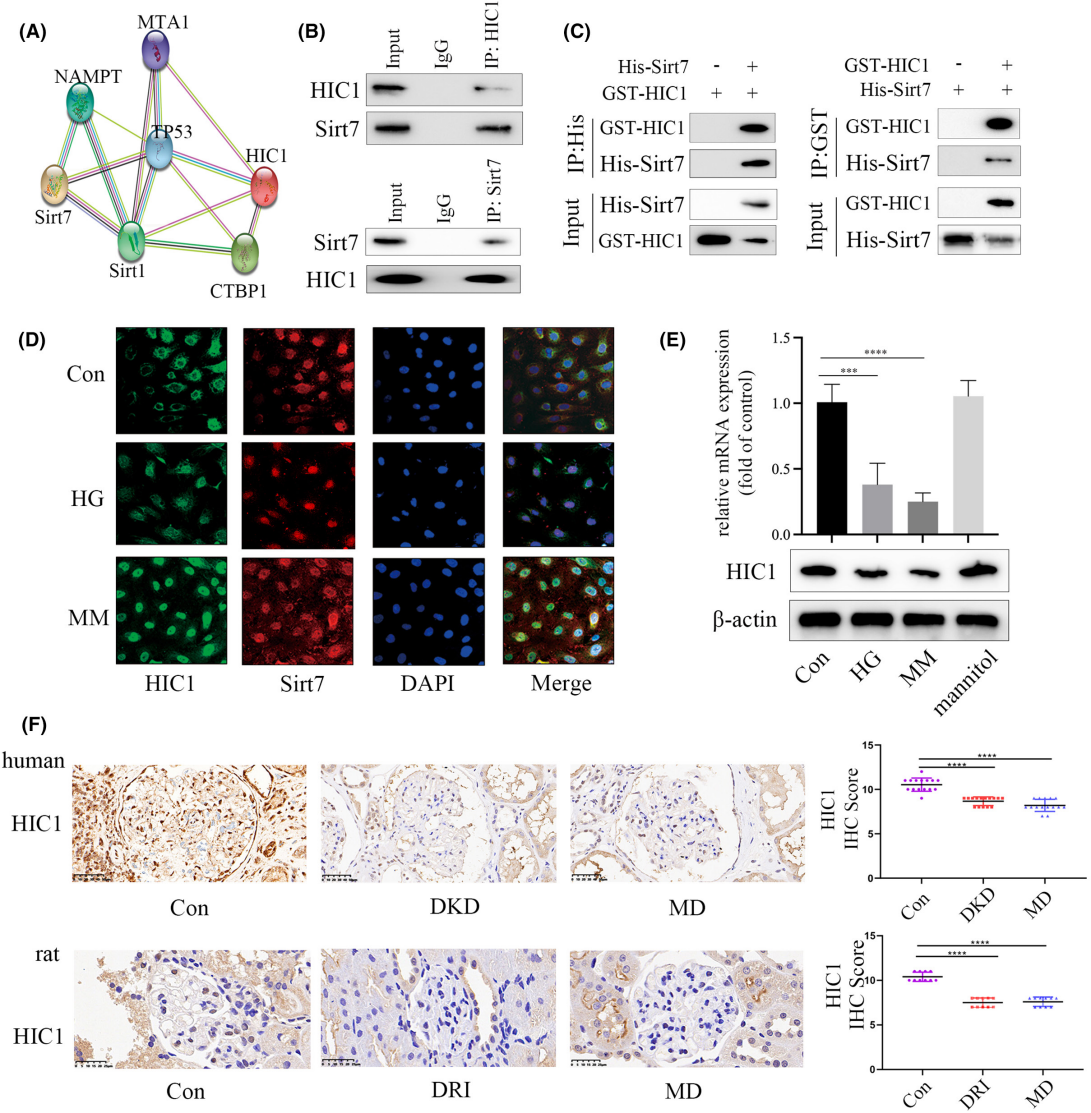
Sirt7 interacted directly with HIC1. (A) A mount of molecules, including HIC1, that may be associated with Sirt7 was predicted via bioinformatics. (B) CoIP assay indicated that Sirt7 interacted with HIC1 in HGECs. (C) GST pull‐down assay indicated that Sirt7 directly associated with HIC1 in HGECs. (D) IF assay indicated that Sirt7 and HIC1 co‐localized in HGECs. Moreover, hyperglycaemia‐mediated HIC1 nuclear translocation persisted even after glucose normalization in HGECs. (E) Results from the Western Blot analysis and qPCR analysis indicated that the reduction of HIC1 expression persisted even after glucose normalization in HGECs. (F) The IHC results of HIC1 in renal biopsy specimens of participants. Our data indicated that the reduction of HIC1 expression persisted even after glycaemia normalization in participants. The IHC results of HIC1 in renal biopsy specimens of rats. Our data indicated that the reduction of HIC1 expression persisted even after glycaemia normalization in glomeruli of the rats. (*p < 0.05, **p < 0.01, ***p < 0.001, ****p < 0.0001, statistical analysis was carried out by a one‐way ANOVA followed by Bonferroni‐corrected pairwise comparisons for over two groups.)

In this study, bioinformatics tools were utilized to uncover the mechanism through which Sirt7 influences the transcription of SDC1 by predicting molecules that interact with Sirt7. Several molecules found to be associated with Sirt7 are depicted in Figure 4A, accessible via the provided link to the InBio Discover platform by Intomics (https://inbio‐discover.intomics.com/map.html#search). This approach highlights the potential interactions and pathways through which Sirt7 may exert its regulatory effects on SDC1 transcription. The association between Sirt7 and HIC1 was further supported by Co‐immunoprecipitation (CoIP) result, as illustrated in Figure 4B, confirming the involvement of HIC1 in modulating EMT, which is consistent with previous study.^26^ The direct interaction between these two proteins was also verified through a GST pull‐down assay, with findings presented in Figure 4C. Immunofluorescence studies confirmed the colocalization of Sirt7 and HIC1 within HGECs, as shown in Figure 4D. Additionally, it was observed that the nuclear translocation of HIC1, induced by high glucose conditions, remained even after the glucose levels were normalized in HGECs, as depicted in Figure 4D. Moreover, a persistent decrease in HIC1 expression was noted both in vivo and in vitro, even after the normalization of glucose levels (Figure 4E,F).

### 3.5 HIC1 participated in hyperglycaemia‐induced EndMT via modulation of SDC1 expression in HGECs

**FIGURE 5:**
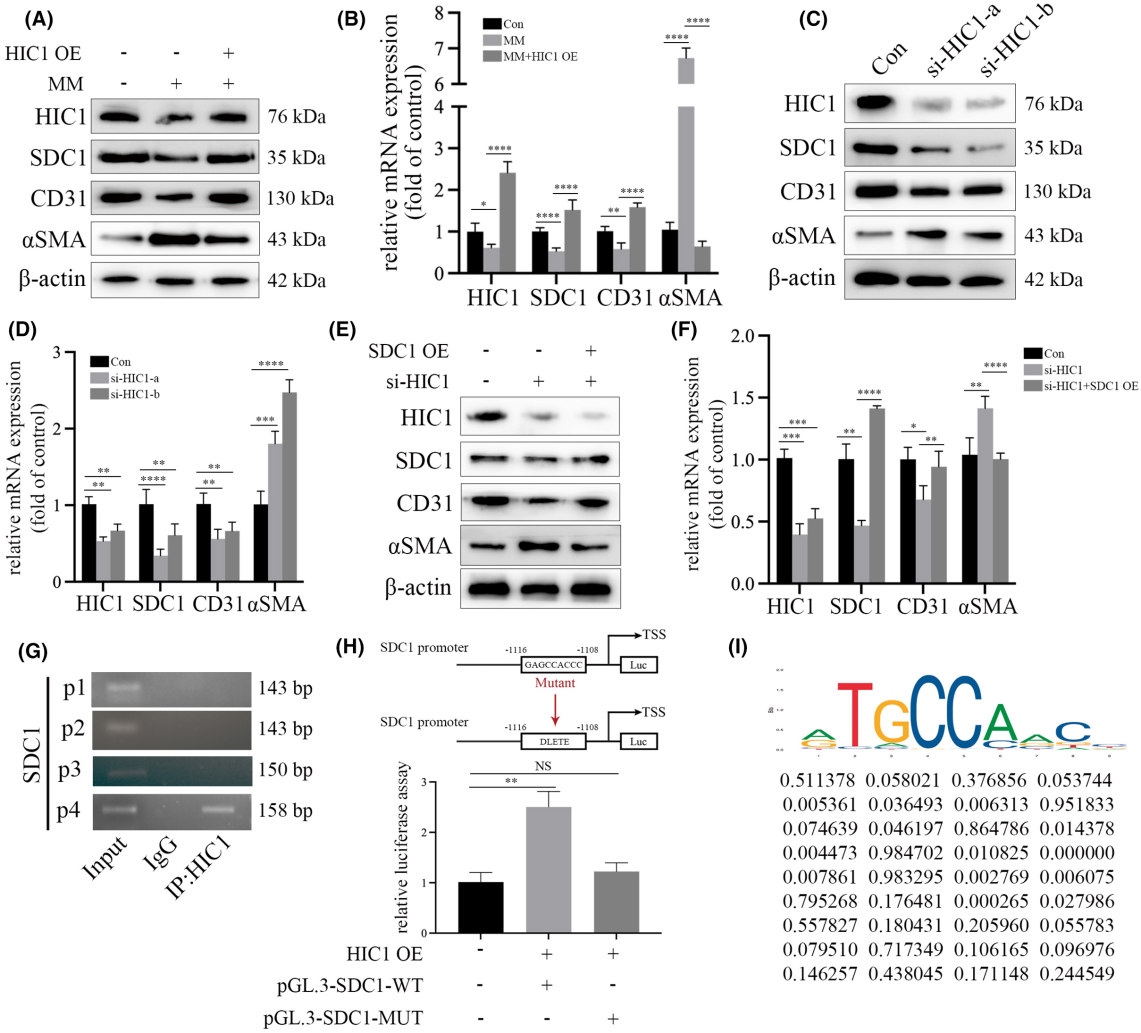
HIC1 participated in hyperglycaemia‐induced EndMT via modulation of SDC1 expression in HGECs. (A) Results from the Western Blot analysis indicated that upregulation of HIC1 expression increased CD31 and SDC1 expression, and decreased αSMA levels in MM‐treated HGECs. (B) Results from the qPCR analysis indicated that overexpression of HIC1 expression increased CD31 and SDC1 expression, and decreased αSMA levels in MM‐treated HGECs. (C) Results from the Western Blot analysis indicated that si‐HIC1 decreased SDC1 and CD31 expression, and increased αSMA levels in HGECs. (D) Results from the qPCR analysis indicated that si‐HIC1 decreased SDC1 and CD31 expression, and increased αSMA levels in HGECs. (E) Results from the Western Blot analysis indicated that the effect of si‐HIC1 on SDC1, CD31 and αSMA expression was reversed via upregulation of SDC1 expression in HGECs. (F) Results from the qPCR analysis indicated that the effect of si‐HIC1 on SDC1, CD31 and αSMA expression was reversed by upregulating SDC1 expression in HGECs. (G) ChIP assay indicated that HIC1 was enriched on the promotor region of SDC1. (H) SDC1 promoter activity was determined by luciferase reporter assays with corresponding treatment. (I) The predicted HIC1 binding site on the promotor region of SDC1. (*p < 0.05, **p < 0.01, ***p < 0.001, ****p < 0.0001, statistical analysis was carried out by a one‐way ANOVA followed by Bonferroni‐corrected pairwise comparisons for over two groups.)

To determine whether HIC1 was involved in hyperglycaemia‐induced metabolic memory and EndMT, both gain‐of‐function and loss‐of‐function approaches were utilized. The results showed the reversed MM‐mediated SDC1 reduction and EndMT in HGECs by overexpression of HIC1 (Figure 5A,B), and si‐HIC1 inhibited SDC1 and exerted a mediative role on EndMT in HGECs, which was similar to that of HG and MM group (Figure 5C,D). The effect of si‐HIC1 was reversed by SDC1 overexpression in HGECs (Figure 5E,F). Further, ChIP assay indicated the enrichment of HIC1 on the promotor of SDC1 (Figure 5G). Luciferase analysis was employed to pinpoint the precise binding site of HIC1 on the DNA sequence, which was identified between −1108 and −1116 base pairs. Mutation of this binding site sequence led to the disappearance of HIC1's enhancing effect on the luciferase reporter activity, as demonstrated in Figure 5H. Additionally, Figure 5I displays both the predicted HIC1 binding site on the DNA and the locations of the primers used in the study, providing a visual representation of the experimental design and findings.

### 3.6 Sirt7 associated with HIC1 to regulate SDC1 transcription

**FIGURE 6:**
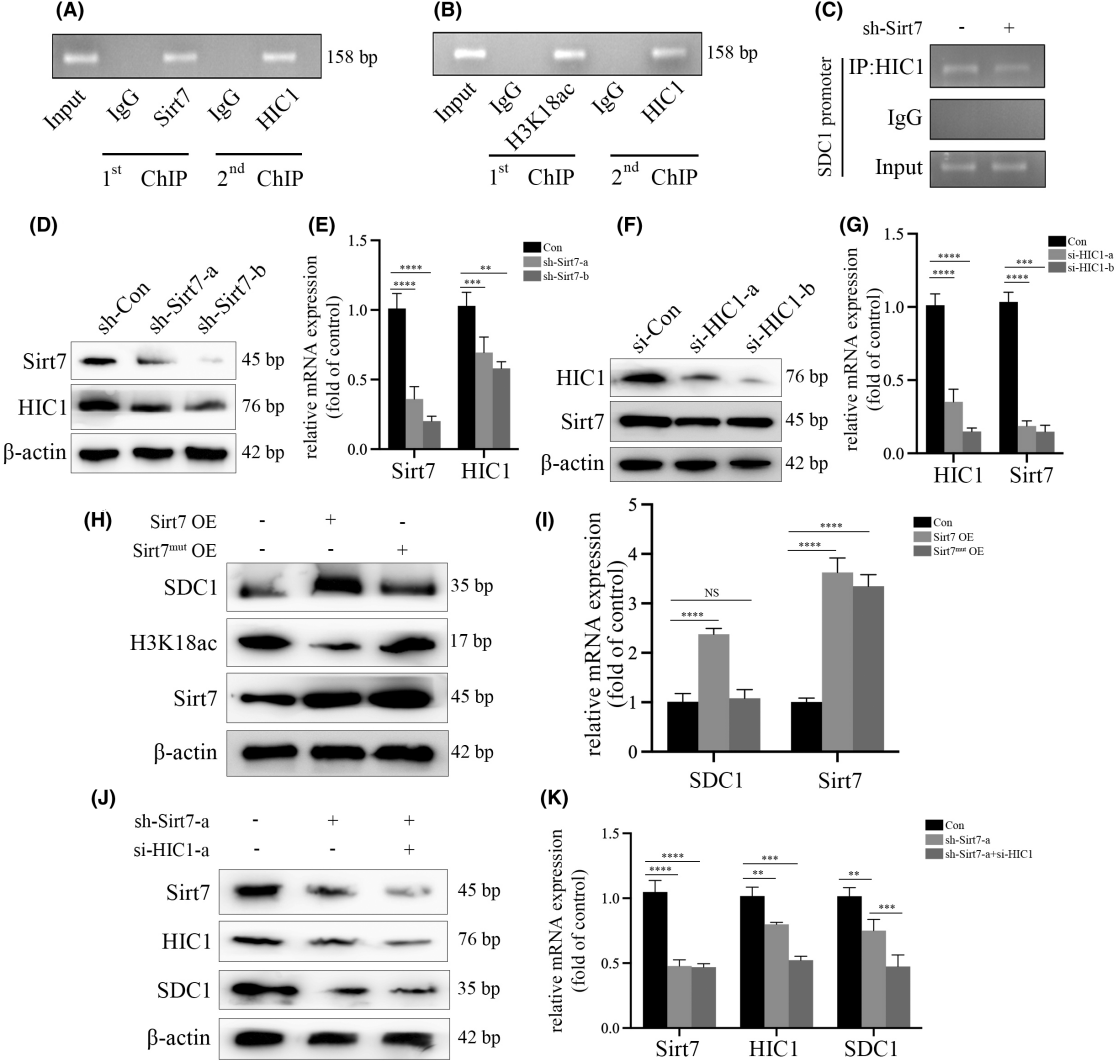
Sirt7 associated with HIC1 to regulate SDC1 transcription. (A) re‐ChIP assay indicated that Sirt7 and HIC1 were enriched on the same promoter region of SDC1 in HGECs. (B) re‐ChIP assay indicated that H3K18ac and HIC1 were enriched on the same promoter region of SDC1 in HGECs. (C) ChIP assay indicated that the enrichment of HIC1 on the promoter region of SDC1 was decreased by sh‐Sirt7 in HGECs. (D) Results from the Western Blot analysis indicated that sh‐Sirt7 decreased HIC1 levels in HGECs. (E) Results from the qPCR analysis indicated that sh‐Sirt7 decreased HIC1 levels in HGECs. (F) Results from the Western Blot analysis indicated that si‐HIC1 decreased Sirt7 levels in HGECs. (G) Results from the qPCR analysis indicated that si‐HIC1 decreased Sirt7 levels in HGECs. (H) Results from the Western Blot analysis indicated that Sirt7 overexpression increased SDC1 expression. However, Sirt7 mutant overexpression had no effect on SDC1 expression. (I) Results from the qPCR analysis indicated that Sirt7 overexpression increased SDC1 expression. However, Sirt7 mutant overexpression had no effect on SDC1 expression. (J) Results from the Western Blot analysis indicated that the effect of sh‐Sirt7 on SDC1 expression was aggravated via si‐HIC1 expression in HGECs. (K) Results from the qPCR analysis indicated that the effect of sh‐Sirt7 on SDC1 expression was aggravated via si‐HIC1 expression in HGECs. (*p < 0.05, **p < 0.01, ***p < 0.001, ****p < 0.0001, statistical analysis was carried out by a one‐way ANOVA followed by Bonferroni‐corrected pairwise comparisons for over two groups.)

According to the outcomes of re‐ChIP assay, Sirt7 and HIC1 were enriched on the same region of SDC1 (Figure 6A,B). And silencing Sirt7 led to a reduction of enrichment of HIC1 (Figure 6C). Furthermore, sh‐Sirt7 could inhibit the expression of HIC1 in HGECs (Figure 6D,E), and si‐HIC1 down‐regulated Sirt7 expression (Figure 6F,G). These results indicated the mediation of high glucose mutual modulation between HIC1 and Sirt7, to form the positive feedback to participate in metabolic memory in DKD. Furthermore, Sirt7 regulated SDC1 transcription and induced EndMT by cooperating with HIC1 in high glucose condition, even after glucose normalization in HGECs. In addition, the SDC1 expression was promoted by Sirt7 overexpression, while Sirt7 mutant (Does not have deacetylation activity) exerted no effect (Figure 6H,I). These results indicated that Sirt7 modulate the SDC1 transcript via modification of H3K18ac in HGECs. In our study, sh‐Sirt7 was found to not only downregulate SDC1 protein and mRNA expression, but also Influence the action of HIC1 on SDC1 levels (Figure 6J,K). All above illustrated that Sirt7 regulated SDC1 transcription via the association with HIC1 to in hyperglycaemic HGECs.

### 3.7 Sirt7 overexpression improved pathological process of metabolic memory and DKD in vivo

**FIGURE 7:**
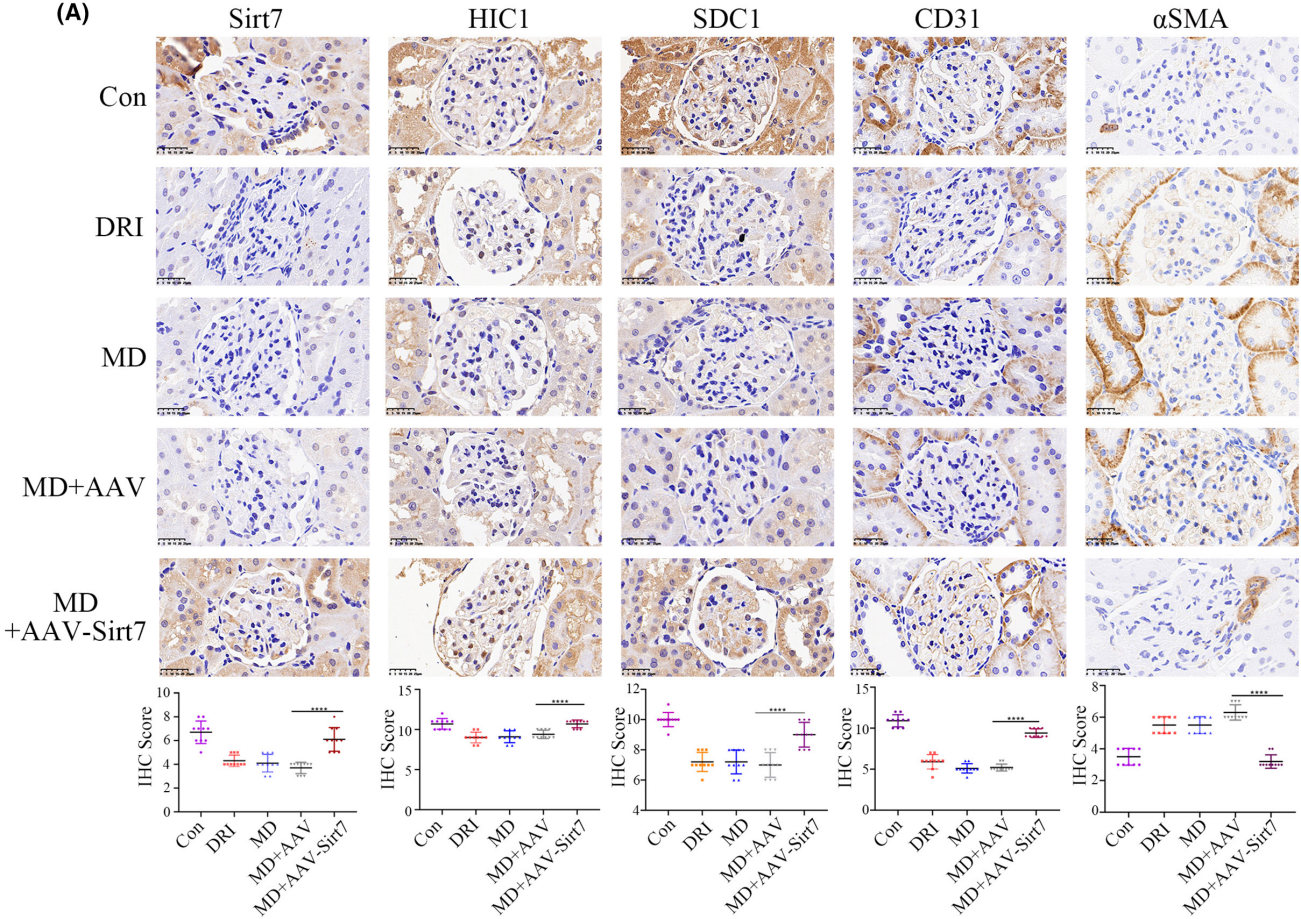
Sirt7 overexpression improved pathological process of DKD in vivo. (A) The IHC results of Sirt7 overexpression associated with glycaemia normalization on the levels of Sirt7, HIC1, SDC1, CD31, αSMA in renal biopsy specimens of rats. Our data indicated that the overexpression of Sirt7 expression associated with glycaemia normalization reversed hyperglycaemia‐mediated EndMT and the reduced levels of HIC1 and SDC1 in glomeruli of the rats. (*p < 0.05, **p < 0.01, ***p < 0.001, ****p < 0.0001, statistical analysis was carried out by a one‐way ANOVA followed by Bonferroni‐corrected pairwise comparisons for over two groups.)

To confirm the protective function of Sirt7 in vivo, the effect of AAV‐Sirt7 was confirmed via IHC (Figure 7A). Our data indicated a downregulation of hyperglycaemia‐induced αSMA by Sirt7 overexpression supplemented with glucose normalization, which increased the hyperglycaemia‐mediated reduction of HIC1, SDC1 and CD31 expression in the kidneys of the rats, while insulin alone did not work (Figure 7A). Enhancing Sirt7 expression, combined with the normalization of glucose levels, led to an improvement in renal dysfunction in rats, a result not achieved by insulin treatment alone, as shown in Figures S2A–K. This evidence suggests that elevating Sirt7 levels ameliorates renal dysfunction in cases of diabetes‐induced renal injury, primarily through the inhibition of EndMT. Additionally, it was observed that increased Sirt7 levels disrupted the positive feedback loop between Sirt7 and HIC1, effectively terminating the persistence of metabolic memory associated with diabetic conditions. To conclude, this study validated the involvement of Sirt7 and HIC1 in metabolic memory in DKD, which mediated EndMT via regulation of SDC1 transcription.

## 4 DISCUSSION

This study revealed that normalization of blood glucose levels did not prevent the onset of diabetic kidney disease (DKD) in both patients and diabetes‐induced kidney injury rat. Furthermore, we elucidated the potential mechanism wherein the Sirt7/HIC1/SDC1 axis influences the EndMT process, thereby contributing to the development of metabolic memory and the progression of DKD.

Our Masson staining results showed the collagen deposition and fibrosis in DKD and MD participants, which is consistent to previous studies.^6^, ^7^, ^8^ The decreased CD31 expression and the augmented αSMA expression in glomeruli of DKD and MD participants and diabetes‐induced renal injury models validated the involvement of hyperglycaemia‐mediated EndMT in the occurrence of metabolic memory and DKD. Previous study indicated a crucial role of SDC1 in regulating EMT.^12^, ^13^ Here, the decreased SDC1 expression was validated both in vivo and in vitro experiments, and knockdown of SDC1 decreased CD31 expression and increased αSMA expression in HGECs. Meanwhile, the overexpression of SDC1 counteracted the effect of MM treatment in HGECs. All above indicated a progressive role of hyperglycaemia in EndMT by inhibiting SDC1 expression, thus participating in the occurrence of metabolic memory and DKD.

As one of the key factors in promoting metabolic memory,^3^ epigenetic modification has also been reported to exert a role of DKD development.^5^ Emerging evidence have implicated the associated of DNA methylation with metabolic memory and the progression of diabetic complications.^27^ We previously demonstrated that histone lysine methylation plays crucial roles in the development of DKD.^16^, ^17^, ^18^, ^19^ Recently, histone acetylation has been demonstrated to exert a prominent function in metabolic memory^20^ and DKD.^21^ Sirt7, with the capacity of histone H3K18 selective deacetylation, has been indicated to exert a regulatory role in epithelial‐to‐mesenchymal transition.^24^ Fatema et al. demonstrated that Sirt7‐dependent deacetylation of PPARγ at K382 promoted lipogenesis in adipocytes.^28^ However, whether and how Sirt7 modulates hyperglycaemia‐mediated EndMT in metabolic memory and DKD remains to be explored.

Herein, we demonstrated that Sirt7 was decreased in glomeruli of DKD and MD patients and diabetes‐induced renal injury rats, as well as in HGECs cultured in HG and MM conditions. Sirt7, together with SIRT1 and SIRT6, are mainly distributed in the nucleus.^29^ Nevertheless, plenty of studies on Sirt7 failed to show the exact subcellular location,^23^, ^24^, ^25^ the whole cell extracts of Sirt7 were used in our experiments. We hypothesized that Sirt7 may exert its function both in the nucleus and in the cytoplasm, which requires further validation. Then we found that the knockdown effect of Sirt7 was reversed by SDC1 overexpression in vitro. These results together demonstrated that Sirt7 participated in hyperglycaemia‐induced EndMT via modulation of SDC1 transcription, to play a role in metabolic memory and DKD.

Sirt7 regulates SDC1 transcription by association with specific transcription factors. Therefore, we carried out bioinformatics, CoIP and IF tests to predict and determine the potential transcription factors interacted with Sirt7. The results demonstrated HIC1 as one of the potential transcription factors. HIC1 is a tumour suppressor gene located at chromosome 17p13.3, and has been reported to induce endothelial to mesenchymal transition with depletion.^26^ However, the underlying mechanism that HIC1 regulates hyperglycaemia‐mediated EndMT in metabolic memory and DKD lacks exploration. Interestingly, HIC1 has been found serve as a target of miR‐4449, and inhibited pyroptosis in DKD patients.^30^ Our in vivo and in vitro experiments showed that HIC1 was downregulated in glomeruli of DKD and MD patients, as well as HGECs cultured in HG and MM conditions. Moreover, inhibition of HIC1 expression mediated EndMT in HGECs, while the overexpression led to the opposite. Furthermore, the knockdown of HIC1 could reach an efficiency similar to MM treatment, which could be reversed by SDC1 overexpression in vitro. These results together illustrated the involvement of HIC1 in hyperglycaemia‐induced EndMT via modulation of SDC1 in metabolic memory and DKD.

It has been reported that the transcriptional activity of HIC1 was mediated by epigenetic modifications.^31^, ^32^ Our results demonstrated an association of Sirt7 with HIC1 in HGECs. Moreover, re‐Chip assay showed the consistent position of Sirt7 and HIC1 on SDC1 promotor. Sirt7 downregulation reduced the enrichment of HIC1 on the promotor of SDC1. The findings of this study demonstrate that Sirt7 engaged with HIC1 to influence SDC1 transcription, thereby facilitating EndMT in HGECs. Furthermore, an increase in Sirt7 expression was associated with an increase in SDC1 expression.

This research is subject to certain limitations. Various cell types such as glomerular podocytes, endothelial cells, mesangial cells, pericytes, fibroblasts, neutrophils, and macrophages constitute key components of kidney tissue, with their interplay being crucial for the microvascular pathophysiological processes.^33^ Endothelial cell dysfunction is notably implicated in the advancement of DKD.^34^ In our experiments, we solely utilized human glomerular endothelial cells to construct an in vitro model of high glucose and metabolic memory, drawing on insights from prior research. The distinct contributions of other cell types and their interactions warrant further examination. Additionally, the reciprocal regulation mechanism between Sirt7 and HIC1 need deeper exploration.

To encapsulate, our findings suggest that hyperglycaemia triggers a mutual regulation between Sirt7 and HIC1 that persists even after glucose levels normalize, thereby establishing a positive feedback loop that contributes to the development of metabolic memory. Moreover, Sirt7, in conjunction with HIC1, regulates SDC1 transcription and the EndMT process even after the restoration of normal glucose levels in HGECs, thereby influencing the onset and progression of DKD.

## AUTHOR CONTRIBUTIONS

**Lihong Lu:** Data curation (equal); formal analysis (equal); project administration (equal). **Minmin Zhu:** Data curation (equal); formal analysis (equal); project administration (equal); resources (equal); software (equal); validation (equal). **Qichao Wu:** Formal analysis (equal); methodology (equal); project administration (equal); resources (equal); software (equal). **Zhirong Sun:** Investigation (equal); methodology (equal); supervision (equal); validation (equal); writing – review and editing (equal). **Xiangyuan Chen:** Conceptualization (equal); project administration (equal); writing – review and editing (lead). **Changhong Miao:** Conceptualization (equal); funding acquisition (equal); supervision (equal); writing – review and editing (equal).

## FUNDING INFORMATION

This work was supported by the National Science Foundation of China (No. 82072213) Shanghai Sailing Program (No. 20YF1407800).

## CONFLICT OF INTEREST STATEMENT

The authors declare that there are no conflicts of interest.

## CONSENT FOR PUBLICATION

All authors have provided consent for publication.

## Supporting information


Figure S1

Figure S2

Table S1

## Data Availability

The data that support the findings of this study are available on request from the corresponding author. The data are not publicly available due to privacy or ethical restrictions.

## References

[1] Packham DK , Alves TP , Dwyer JP , et al. Relative incidence of ESRD versus cardiovascular mortality in proteinuric type 2 diabetes and nephropathy: results from the DIAMETRIC (diabetes mellitus treatment for renal insufficiency consortium) database. Am J Kidney Dis. 2012;59:75‐83. 22051245 10.1053/j.ajkd.2011.09.017

[2] Tomino Y , Gohda T . The prevalence and management of diabetic nephropathy in Asia. Kidney Dis. 2015;1(1):52‐60. 10.1159/000381757 PMC4934822 27536665

[3] Reddy MA , Zhang E , Natarajan R . Epigenetic mechanisms in diabetic complications and metabolic memory. Diabetologia. 2015;58:443‐455. 25481708 10.1007/s00125-014-3462-y PMC4324095

[4] Liakishev AA . [Intensive diabetes treatment and cardiovascular disease in patients with type 1 diabetes. Results of the DCCT/EDIC study]. Kardiologiia. 2006;46(3):73. 16710261

[5] Keating ST , El‐Osta A . Glycemic memories and the epigenetic component of diabetic nephropathy. Curr Diab Rep. 2013;13(4):574‐581. 23639991 10.1007/s11892-013-0383-y

[6] Yao Y , Song Q , Hu C , et al. Endothelial cell metabolic memory causes cardiovascular dysfunction in diabetes. Cardiovasc Res. 2022;118(1):196‐211. 33483741 10.1093/cvr/cvab013

[7] Li J , Qu X , Yao J , et al. Blockade of endothelial mesenchymal transition by a Smad3 inhibitor delays the early development of streptozotocin‐induced diabetic nephropathy. Diabetes. 2010;59(10):2612‐2624. 20682692 10.2337/db09-1631 PMC3279546

[8] Kanasaki K , Shi S , Kanasaki M , et al. Linagliptin mediated DPP‐4 inhibition ameliorates kidney fibrosis in streptozotocin induced diabetic mice by inhibiting endothelial‐to‐mesenchymal transition in a therapeutic regimen. Diabetes. 2014;63(6):2120‐2131. 24574044 10.2337/db13-1029

[9] Liang X , Duan N , Wang Y , et al. Advanced oxidation protein products induce endothelial‐to‐mesenchymal transition in human renal glomerular endothelial cells through induction of endoplasmic reticulum stress. J Diabetes Complications. 2016;30(4):573‐579. 26861949 10.1016/j.jdiacomp.2016.01.009

[10] Saito A . EMT and EndMT: regulated in similar ways. Comment J Biochem. 2013;153(6):493‐495. 23613024 10.1093/jb/mvt032

[11] Szatmari T , Otvos R , Hjerpe A , Dobra K . Syndecan‐1 in cancer: implications for cell signaling, differentiation, and prognostication. Dis Markers. 2015;2015:796052. 26420915 10.1155/2015/796052 PMC4569789

[12] Loussouarn D , Campion L , Sagan C , et al. Prognostic impact of syndecan‐1 expression in invasive ductal breast carcinomas. Br J Cancer. 2008;98(12):1993‐1998. 18542065 10.1038/sj.bjc.6604400 PMC2441962

[13] Wang X , He J , Zhao X , Qi T , Zhang T , Kong C . Syndecan‐1 suppresses epithelial‐mesenchymal transition and migration in human oral cancer cells. Oncol Rep. 2018;39(4):1835‐1842. 29484435 10.3892/or.2018.6271

[14] Kato M , Natarajan R . Epigenetics and epigenomics in diabetic kidney disease and metabolic memory. Nat Rev Nephrol. 2019;15(6):327‐345. 30894700 10.1038/s41581-019-0135-6 PMC6889804

[15] Li X , Lu L , Hou W , et al. Epigenetics in the pathogenesis of diabetic nephropathy. Acta Biochim Biophys Sin. 2022;54(2):1‐10. 10.3724/abbs.2021016 PMC9909329 35130617

[16] Huang T , Li X , Wang F , et al. The CREB/KMT5A complex regulates PTP1B to modulate high glucose‐induced endothelial inflammatory factor levels in diabetic nephropathy. Cell Death Dis. 2021;12(4):333. 33782381 10.1038/s41419-021-03629-4 PMC8005662

[17] Lu L , Zhong Z , Gu J , Nan K , Zhu M , Miao C . ets1 associates with KMT5A to participate in high glucose‐mediated EndMT via upregulation of PFN2 expression in diabetic nephropathy. Mol Med. 2021;27(1):74. 34238215 10.1186/s10020-021-00339-7 PMC8266168

[18] Lu L , Li X , Zhong Z , et al. KMT5A downregulation participated in high glucose‐mediated EndMT via upregulation of ENO1 expression in diabetic nephropathy. Int J Biol Sci. 2021;17(15):4093‐4107. 34803485 10.7150/ijbs.62867 PMC8579450

[19] Hou W , Lu L , Li X , Sun M , Zhu M , Miao C . c‐Myc participates in high glucose‐mediated endothelial inflammation via upregulation of IRAK1 expression in diabetic nephropathy. Cell Signal. 2022;92:110263. 35085772 10.1016/j.cellsig.2022.110263

[20] Zhong Q , Kowluru RA . Role of histone acetylation in the development of diabetic retinopathy and the metabolic memory phenomenon. J Cell Biochem. 2010;110(6):1306‐1313. 20564224 10.1002/jcb.22644 PMC2907436

[21] Li X , Li C , Sun G . Histone acetylation and its modifiers in the pathogenesis of diabetic nephropathy. J Diabetes Res. 2016;2016:4065382. 27379253 10.1155/2016/4065382 PMC4917685

[22] Michishita E , Park JY , Burneskis JM , Barrett JC , Horikawa I . Evolutionarily conserved and nonconserved cellular localizations and functions of human SIRT proteins. Mol Biol Cell. 2005;16(10):4623‐4635. 16079181 10.1091/mbc.E05-01-0033 PMC1237069

[23] Li L , Shi L , Yang S , et al. SIRT7 is a histone desuccinylase that functionally links to chromatin compaction and genome stability. Nat Commun. 2016;7:12235. 27436229 10.1038/ncomms12235 PMC4961794

[24] Li W , Zhu D , Qin S . SIRT7 suppresses the epithelial‐to‐mesenchymal transition in oral squamous cell carcinoma metastasis by promoting SMAD4 deacetylation. J Exp Clin Cancer Res. 2018;37(1):148. 30001742 10.1186/s13046-018-0819-y PMC6044017

[25] Wang X , Lin B , Nie L , Li P . microRNA‐20b contributes to high glucose‐induced podocyte apoptosis by targeting SIRT7. Mol Med Rep. 2017;16(4):5667‐5674. 28849008 10.3892/mmr.2017.7224

[26] Wang Y , Weng X , Wang L , et al. HIC1 deletion promotes breast cancer progression by activating tumor cell/fibroblast crosstalk. J Clin Invest. 2018;128(12):5235‐5250. 30204129 10.1172/JCI99974 PMC6264654

[27] Zhao J , Yang S , Shu B , et al. Transient high glucose causes persistent vascular dysfunction and delayed wound healing by the DNMT1‐mediated Ang‐1/NF‐kappaB pathway. J Invest Dermatol. 2021;141(6):1573‐1584. 33259831 10.1016/j.jid.2020.10.023

[28] Akter F , Tsuyama T , Yoshizawa T , Sobuz SU , Yamagata K . SIRT7 regulates lipogenesis in adipocytes through deacetylation of PPARγ2. J Diabetes Investig. 2021;12(10):1765‐1774. 10.1111/jdi.13567 PMC8504911 33955199

[29] Bian C , Ren H . Sirtuin family and diabetic kidney disease. Front Endocrinol (Lausanne). 2022;13:901066. 35774140 10.3389/fendo.2022.901066 PMC9238361

[30] Gao C , Wang B , Chen Q , Wang M , Fei X , Zhao N . Serum exosomes from diabetic kidney disease patients promote pyroptosis and oxidative stress through the miR‐4449/HIC1 pathway. Nutr Diabetes. 2021;11(1):33. 34732690 10.1038/s41387-021-00175-y PMC8566490

[31] Li P , Liu X , Dong ZM , Ling ZQ . Epigenetic silencing of HIC1 promotes epithelial‐mesenchymal transition and drives progression in esophageal squamous cell carcinoma. Oncotarget. 2015;6(35):38151‐38165. 26510908 10.18632/oncotarget.5832 PMC4741990

[32] Zheng J , Wang J , Sun X , et al. HIC1 modulates prostate cancer progression by epigenetic modification. Clin Cancer Res. 2013;19(6):1400‐1410. 23340301 10.1158/1078-0432.CCR-12-2888

[33] Zhang K , Fu Z , Zhang Y , Chen X , Cai G , Hong Q . The role of cellular crosstalk in the progression of diabetic nephropathy. Front Endocrinol (Lausanne). 2023;14:1173933. 37538798 10.3389/fendo.2023.1173933 PMC10395826

[34] Cheng H , Harris RC . Renal endothelial dysfunction in diabetic nephropathy. Cardiovasc Hematol Disord Drug Targets. 2014;14(1):22‐33. 24720460 10.2174/1871529x14666140401110841 PMC4657140

