# Supplementary figures and images for: Sirt7/HIC1 complex participates in hyperglycaemia‐mediated EndMT via modulation of SDC1 expression in diabetic kidney disease and metabolic memory
Source: J Cell Mol Med. 2024 Apr 30;28(9):e18336. doi: 10.1111/jcmm.18336 (PMC11058670; doi:10.1111/jcmm.18336)

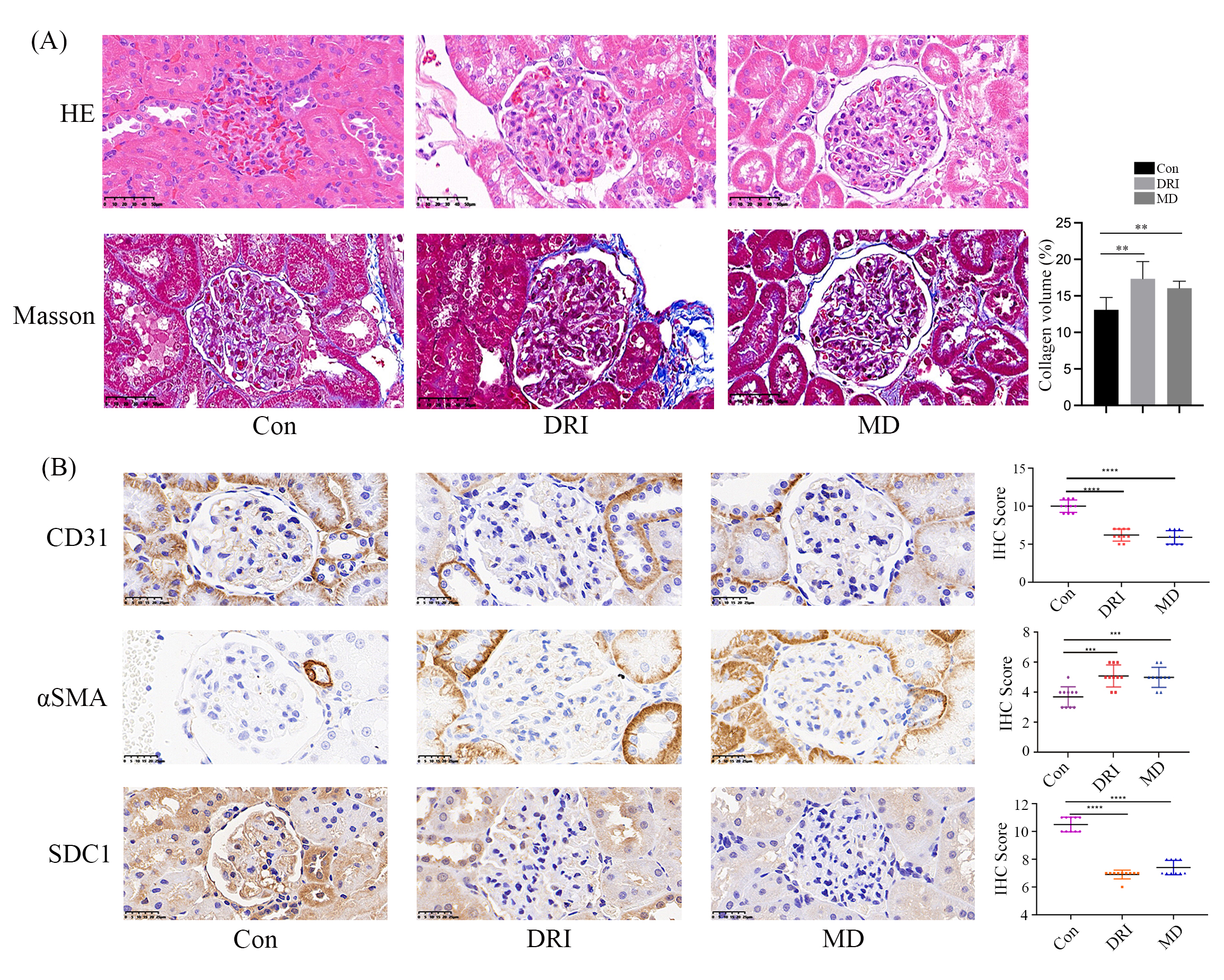

Supplement: Supplementary file 1 — Figure S1 [file JCMM-28-e18336-s001.jpg]

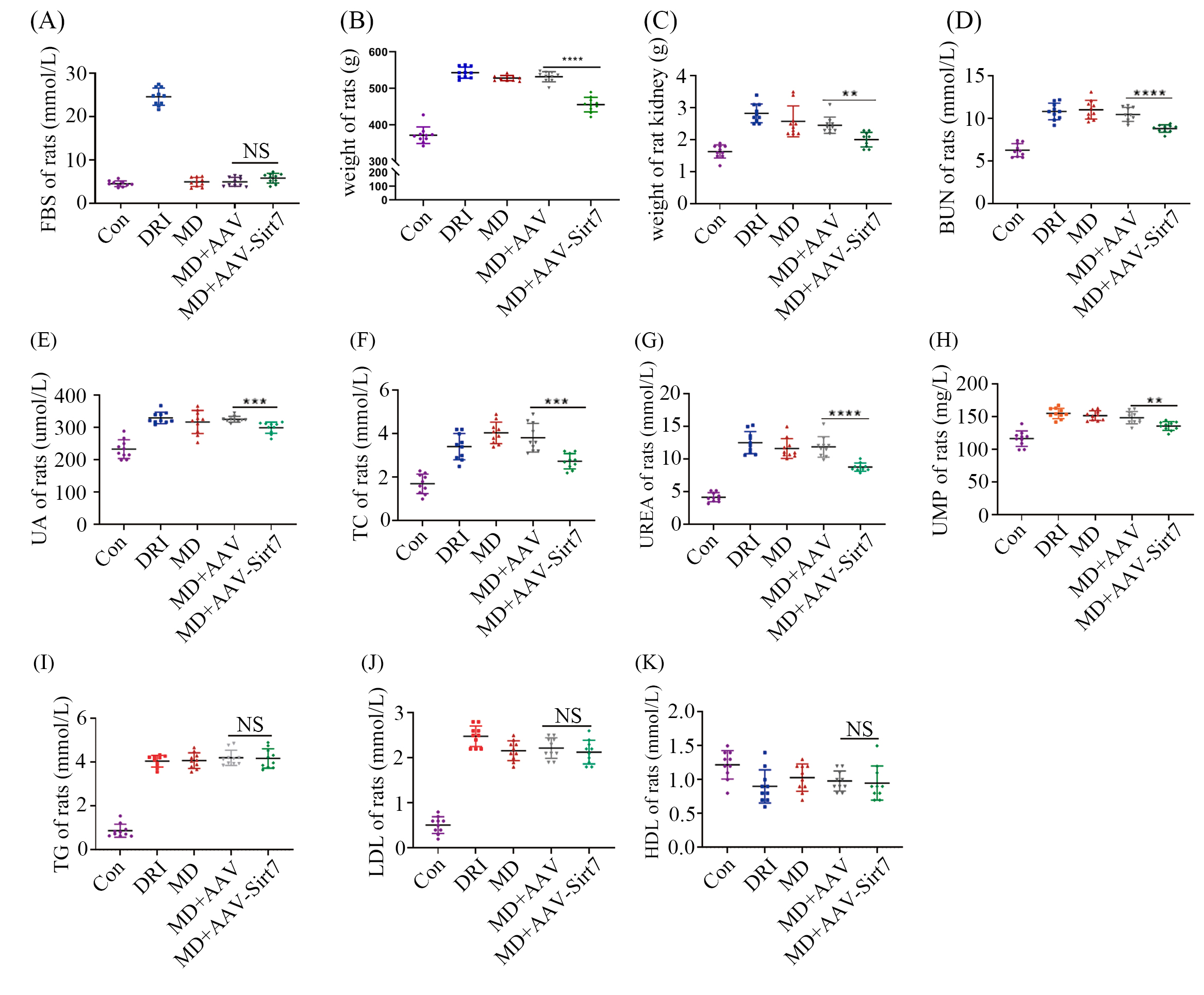

Supplement: Supplementary file 2 — Figure S2 [file JCMM-28-e18336-s002.jpg]

Supplementary Table 1 Primers used for real-time RT-PCR analysis.


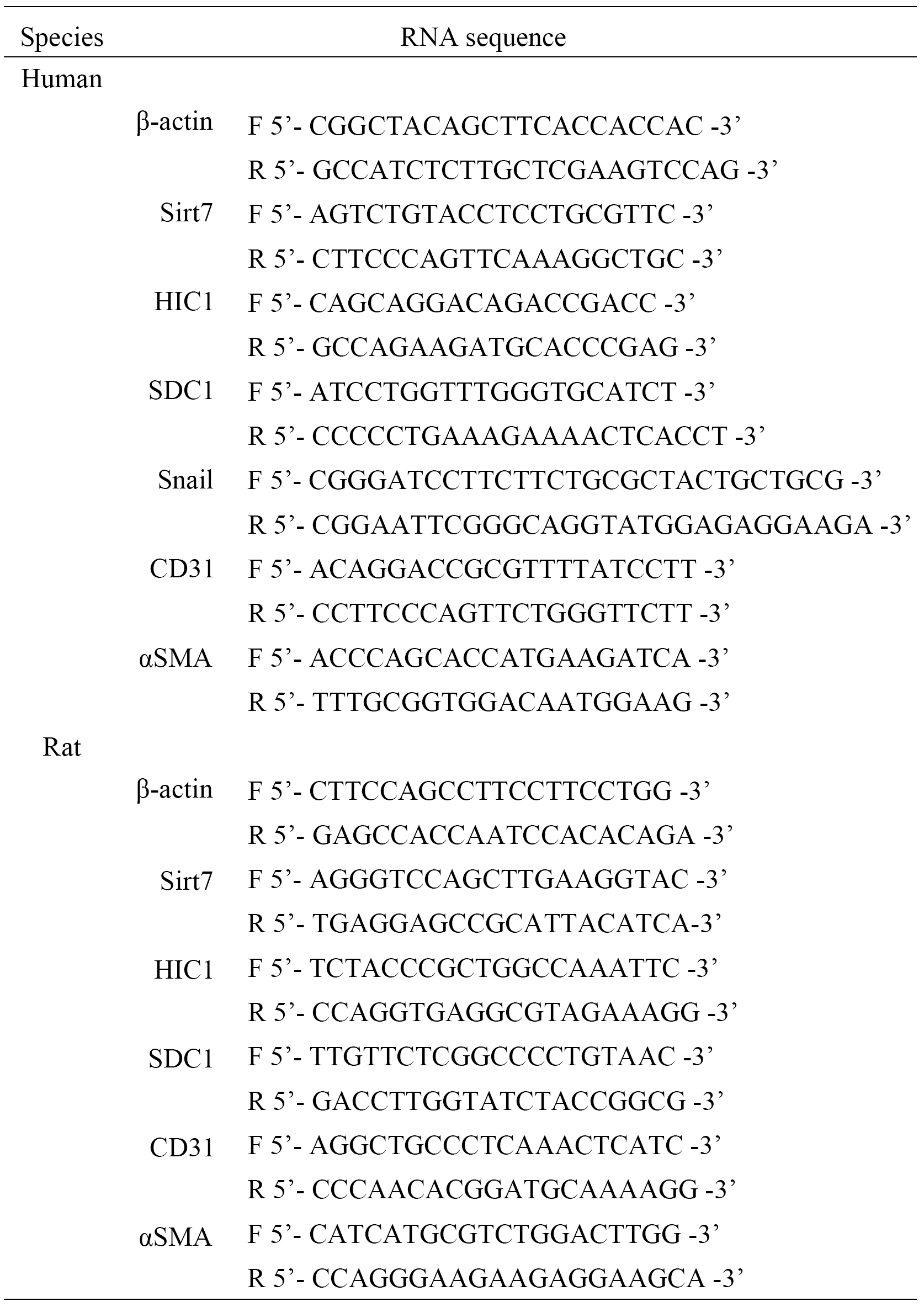

Supplement: Supplementary file 3 — Table S1 [file JCMM-28-e18336-s003.docx]
